# 209. Validation of a New Risk Stratification System-Based Management for Methicillin-Resistant *Staphylococcus aureus* Bacteremia: Analysis of a Multicenter Prospective Study

**DOI:** 10.1093/ofid/ofad500.282

**Published:** 2023-11-27

**Authors:** Taeeun Kim, Sang-Rok Lee, Seong Yeon Park, Song Mi Moon, Jiwon Jung, Min Jae Kim, Heungsup Sung, Mi-Na Kim, Sung-Han Kim, Sang-Ho Choi, Sang-Oh Lee, Yang Soo Kim, Eun Hee Song, Yong Pil Chong

**Affiliations:** Nowon Eulji University Hospital, Seoul, Seoul-t'ukpyolsi, Republic of Korea; Cheongju St Mary’s Hospital, Cheongju, Ch'ungch'ong-bukto, Republic of Korea; Dongguk University Ilsan Hospital, Goyang, Kyonggi-do, Republic of Korea; Seoul National University College of Medicine, Seoungnam-si, Kyonggi-do, Republic of Korea; Asan Medical Center, Seoul, Seoul-t'ukpyolsi, Republic of Korea; Asan Medical Center, Seoul, Seoul-t'ukpyolsi, Republic of Korea; Asan Medical Center, Seoul, Seoul-t'ukpyolsi, Republic of Korea; Asan Medical Center, Seoul, Seoul-t'ukpyolsi, Republic of Korea; Asan medical center, Seoul, Seoul-t'ukpyolsi, Republic of Korea; Asan Medical Center, Seoul, Seoul-t'ukpyolsi, Republic of Korea; Asan Medical Center, Seoul, Seoul-t'ukpyolsi, Republic of Korea; Asan Medical Center, Seoul, Seoul-t'ukpyolsi, Republic of Korea; Gangneung Asan Hospital, Gangneung, Kangwon-do, Republic of Korea; Asan Medical Center, Seoul, Seoul-t'ukpyolsi, Republic of Korea

## Abstract

**Background:**

Distinguishing between complicated and uncomplicated *Staphylococcus aureus* bacteremia (SAB) is therapeutically essential. However, this distinction has limitations in reflecting the various manifestation of SAB. In the light of this, Koujizer *et al*. proposed a new risk stratification system for metastatic infection in SAB, which involves a stepwise approach to diagnosis and treatment. We tested this risk stratification system in methicillin-resistant SAB (MRSAB) patients.

**Methods:**

We retrospectively analyzed data of a 3-year multicenter, prospective cohort of hospitalized patients with MRSAB. We classified the patients into three risk groups: low, indeterminate, and high, based on the new system and compared between-group management and outcomes, as well as microbiologic features.

**Results:**

The demographic and baseline characteristics of patients are shown in Table 1. The most frequent source of MRSAB was central venous catheter-related infection (25.5%), followed by unknown origin (15.3%) and pneumonia (11.1%). Echocardiography was performed in 248 cases (65.3%). Of 380 patients with MRSAB, 6.3% were classified as low-, 7.6% as indeterminate-, and 86.1% as high-risk for metastatic infection (Figure 1). No metastatic infection occurred in the low-, 6.9% in the indeterminate-, and 17.7% in the high-risk groups (*P*=0.03) (Figure 2A). After an in-depth diagnostic work-up, patients were finally diagnosed as ‘without metastatic infection (6.3%)’, ‘with metastatic infection (15.8%)’, and ‘uncertain metastatic infection (77.9%)’. 30-day mortality increased markedly as the severity of diagnosis shifted from ‘without metastatic infection’ to ‘uncertain’ and ‘with metastatic infection’ (*P*=0.07) (Figure 2B). In multivariable analysis, independent factors associated with metastatic complications were suspicion of endocarditis in transthoracic echocardiography, clinical signs of metastatic infection, Pitt bacteremia score ≥4, and persistent bacteremia.

**Figure:**
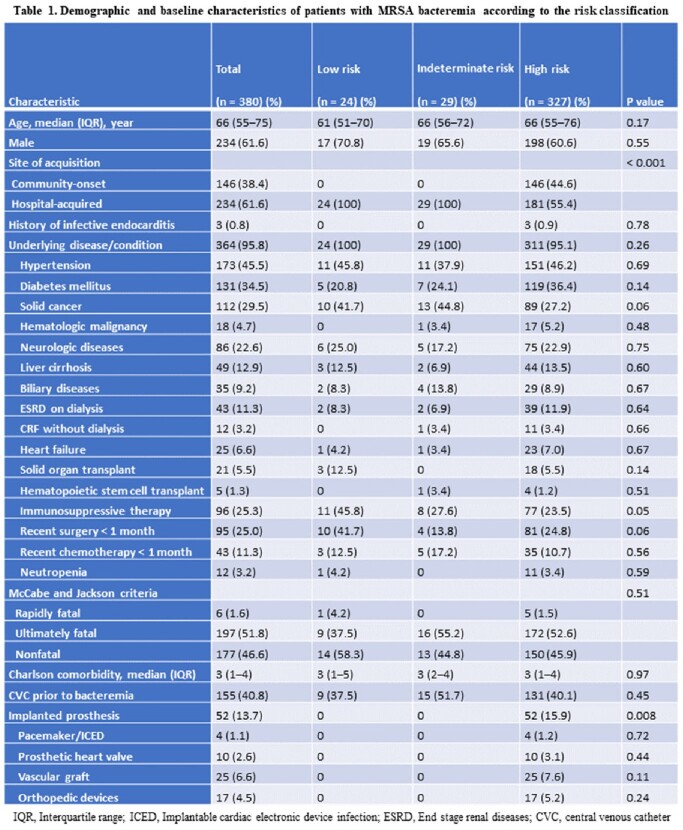

Figure 1.

**Figure:**
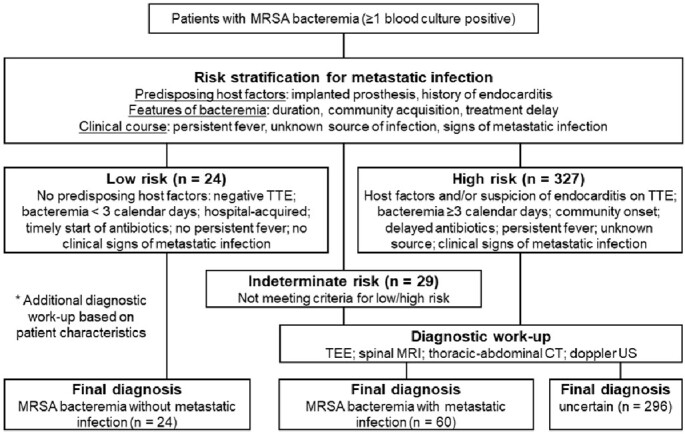

Flowchart of risk stratification and final diagnosis

Figure 2.

**Figure:**
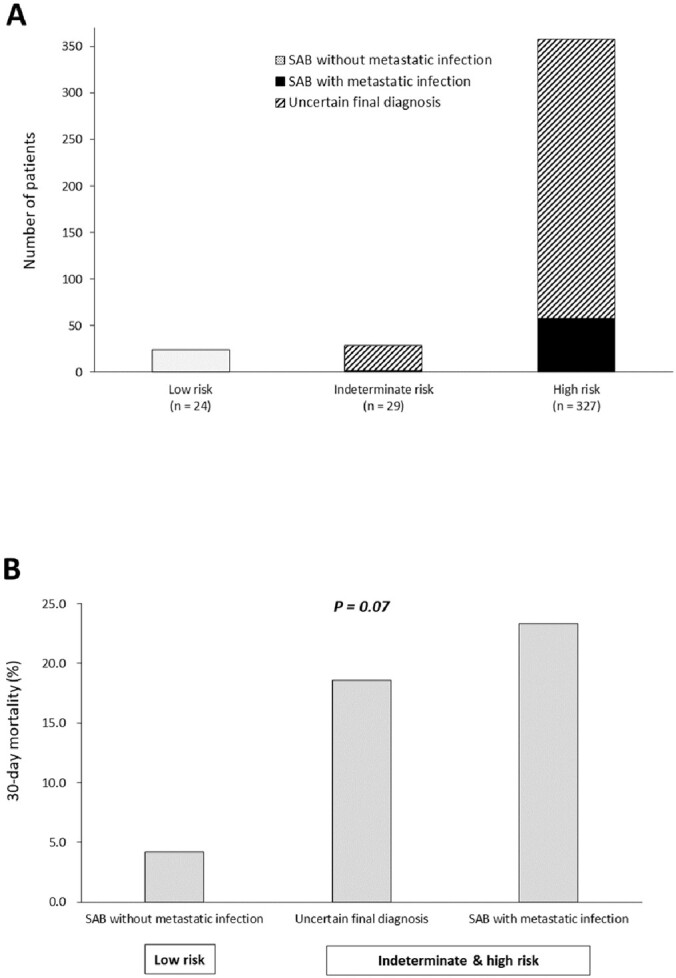

Outcomes of metastatic infection and 30-day mortality. A, metastatic infection according to the risk stratification, B, 30-day mortality according to the final diagnosis

**Conclusion:**

The new risk stratification system provides good discrimination in predicting metastatic complications, making it a reliable tool for guiding work-up and management of MRSAB. However, minimizing the number of ‘uncertain metastatic infection’ cases remains an area for improvement.

**Disclosures:**

**All Authors**: No reported disclosures

